# Comparison of Drug Availability in the Inner Ear After Oral, Transtympanic, and Combined Administration

**DOI:** 10.3389/fneur.2021.641593

**Published:** 2021-08-19

**Authors:** Yang Li, Sho Kanzaki, Shinsuke Shibata, Masaya Nakamura, Masahiro Ozaki, Hideyuki Okano, Kaoru Ogawa

**Affiliations:** ^1^Department of Otolaryngology Head and Neck Surgery, Keio University School of Medicine, Tokyo, Japan; ^2^Department of Otolaryngology Head and Neck Surgery, Second Affiliated Hospital, Xi'an Jiaotong University, Xi'an, China; ^3^Department of Physiology, Keio University School of Medicine, Tokyo, Japan; ^4^Department of Orthopedics, Keio University School of Medicine, Tokyo, Japan

**Keywords:** drug delivery system, inner ear, bioluminescence imaging, mouse, pharmacokinetics

## Abstract

Although combination of oral and transtympanic drug therapy (CT) has been proved more effective and safer for idiopathic sudden sensorineural hearing loss (ISSNHL) by some clinical trials, there are few laboratory researches on the pharmacokinetics in the inner ear following CT on account of structural limitations of the inner ear. The aim of the present study was to investigate the pharmacokinetic behaviors of CT in the inner ear of mice. Eighteen transgenic *GFAP-Luc* mice which express luciferase in cochlear spiral ganglion cells were divided into oral administration (OR) group, transtympanic injection route (TT) group and CT group, and luciferin was delivered into the inner ear of these mice through oral, transtympanic or combined routes, respectively. A new *in vivo* imaging system was used to observe luciferin/luciferase signals and the compare the pharmacokinetics of different administration routes in the inner ear of mice. Bioluminescence signals were observed in the inner ear 3.3 ± 2.6 min after CT, significantly earlier than that of OR group (15.8 ± 7.4 min). CT owned the longest reaching-peak time and largest area under the curve (AUC) among three groups. Compared to TT, CT had longer biological half-life and higher AUC value, but did not displayed stronger peak value. There were significant differences in the peak values between OR group and TT group and between OR group and CT group. This study suggests that the OR route is less effective than the TT or CT route, and combination of OR and TT can deliver more drugs into the inner ear and confer a longer therapeutic window, but cannot increase drug intensity.

## Introduction

Inner ear disorders are major clinical diseases that significantly impact human health. The effective and safe treatment of these diseases has become increasingly dependent on inner ear drug delivery systems. Currently, the oral route of administration (OR) is considered as the first line approach as the treatment modality for inner ear disorders such as sudden idiopathic sensorineural hearing loss (ISSNHL), Meniere's disease and noise-induced hearing loss because of the convenience of administration in the form of pills, but largely ineffective due to the poor penetration of the blood labyrinth barrier (BLB). Many drugs' clinical usefulness is limited by systemic drug toxicity and the adverse effects related to the high doses required to achieve sufficient therapeutic effects (1, 2). The transtympanic route of administration (TT) can bypass the BLB and access directly the inner ear through round window membrane, increasing the drug concentration at the targeted organ and reducing the systemic adverse effects. However, this route requires repeated injections to achieve a prolonged residence in the middle ear and a therapeutic concentration in the inner ear (3, 4). Recently, combination of oral and transtympanic therapy (CT) has become an attractive strategy for inner ear drug delivery (5). Although CT has been proved more effective and safer for ISSNHL by some clinical trials (6–9), and there are few laboratory researches on the pharmacokinetics in the inner ear following combined drug application. For better understanding the mechanism of CT, it is very imperative to investigate its pharmacokinetic behaviors, especially the amount of drug reaching the inner ear and its residence time in the inner ear.

Despite some studies using dissection and direct measurement of drug levels in the perilymph (10, 11), it is impossible to monitor the changes in inner ear pharmacokinetics over time in the same animal on account of the inner ear's anatomical, histological, and structural limitations. To overcome these problems, we recently established a new *in vivo* imaging system (IVIS) to monitor drug delivery to the spiral ganglion cells of the inner ear in live transgenic mice to compare drug concentrations over time after intravenous, transtympanic, and combined injections (12–15). In this system, glial fibrillary acidic protein-luciferase transgene (GFAP-Luc) is expressed in non-myelinating schwann cells in the spiral ganglion, and the enzyme luciferase reacts with its ligand, luciferin, to generate measurable luminescence. In the present study, we assess the pharmacokinetic behaviors of D-luciferin signals in the inner ear of mice following oral, local and combined administration of D-luciferin in real-time for the first time. We expect the results will provide a meaningful suggestion for the use of combined inner ear drug delivery in clinical practice.

## Materials and Methods

### GFAP-Luc Mice

Transgenic *GFAP-Luc* mice were obtained from Xenogen Corporation (Alameda, CA). *GFAP-Luc* mice harbor a firefly luciferase gene expression cassette that is regulated by a 12-kb sequence comprising the murine *GFAP* promoter and intron 2 of the gene that encodes human β-globin 2 (16). Luciferin delivered to these mice's inner ears is oxidized by luciferase expressed by luciferase-expressing cells in the cochlear nerve and the spiral ganglion. A camera was used to detect the emitted photons.

All experiments were approved by and carried out under the Animal Care and Use Committee of Keio University (Permit Number: 08020) following the Guide for the Care and Use of Laboratory Animals (National Institutes of Health, Bethesda, MD, USA).

### Groups

Thirteen *GFAP-Luc* mice aged 6–8 weeks old (body weight: 26–34 g) were divided into three groups based on delivery method: (1) oral administration group (OR, *n* = 6), (2) transtympanic injection group (TT, *n* = 6), (3) combination therapy group (CT, *n* = 6). We removed the auricle to facilitate monitoring of luciferin delivery to the cochlea.

### Oral, Transtympanic, and Combination Drug Delivery

The mice were anesthetized with an intraperitoneal (i.p.) injection of ketamine (100 mg/kg) and xylazine (10 mg/kg) after being deprived of food and drink for 8 h. For OR administration, 280 μl of D-luciferin was administered orally. TT drug delivery was performed securely under a surgical microscope. Two perforations (for ventilation and injection) were made on the first quadrant (anterior upper quadrant) of the left tympanic membrane using a 30-gauge needle, and 70 μl of D-luciferin was injected into the middle ear cavity *via* the injection site. For combination drug delivery, the mice were firstly given 280 μl of oral D-luciferin, then another 70 μl of D-luciferin was injected *via* the tympanic membrane. The concentration of D-luciferin in three groups is 15 mg/ml.

### Bioluminescence Imaging

An IVIS spectrum and a CCD optical macroscopic imaging system (Xenogen, Alameda, CA) were used for spatiotemporal detection of the luciferase-luciferin reaction (12, 13, 17). *In vivo* bioluminescent images were captured immediately the luciferase substrate, D-(-)-2-(6′-hydroxy-2′-benzothiazolyl) thiazone-4-carboxylicacid (D-luciferin) injection, with the field of view set at 10 cm and an integration time of 5 min. All images were analyzed using Living Image software (Xenogen). The optical signal intensity was expressed as photon flux (photon count) in units of photons/s/cm^2^/steradian. Each image was displayed as a pseudocolored photon-count image superimposed onto a grayscale anatomic image of the inner ear. To quantify the measured light, we defined the regions of interest (ROI) in the inner ear and examined all values in that ROI.

We analyzed five parameters: (1) time to inner ear, (2) peak photon count, (3) T_max_ (time-to-peak), (4) T_1/2_ (biological half-life), and (5) area under the curve (AUC). AUC was analyzed using the free software “moment.xls,” downloaded from the Department of Biopharmaceutics and Drug Metabolism, Kyoto University (http://www.pharm.kyoto-u.ac.jp//byoyaku/English/).

### Statistics

Time to the inner ear, peak counts, peak times, half-lives, and AUC in the OR, TT, and CT groups were compared using one-way analysis of variance. The half-life was defined as the time at which the emission time reached 50% of the initial peak value during the acquisition of photon counts. Significant differences among the three groups were analyzed using the Tukey method. All scores were averaged and analyzed using SPSS software 20.0 (IBM Corp., Armonk, NY).

## Results

**Figure 1:**
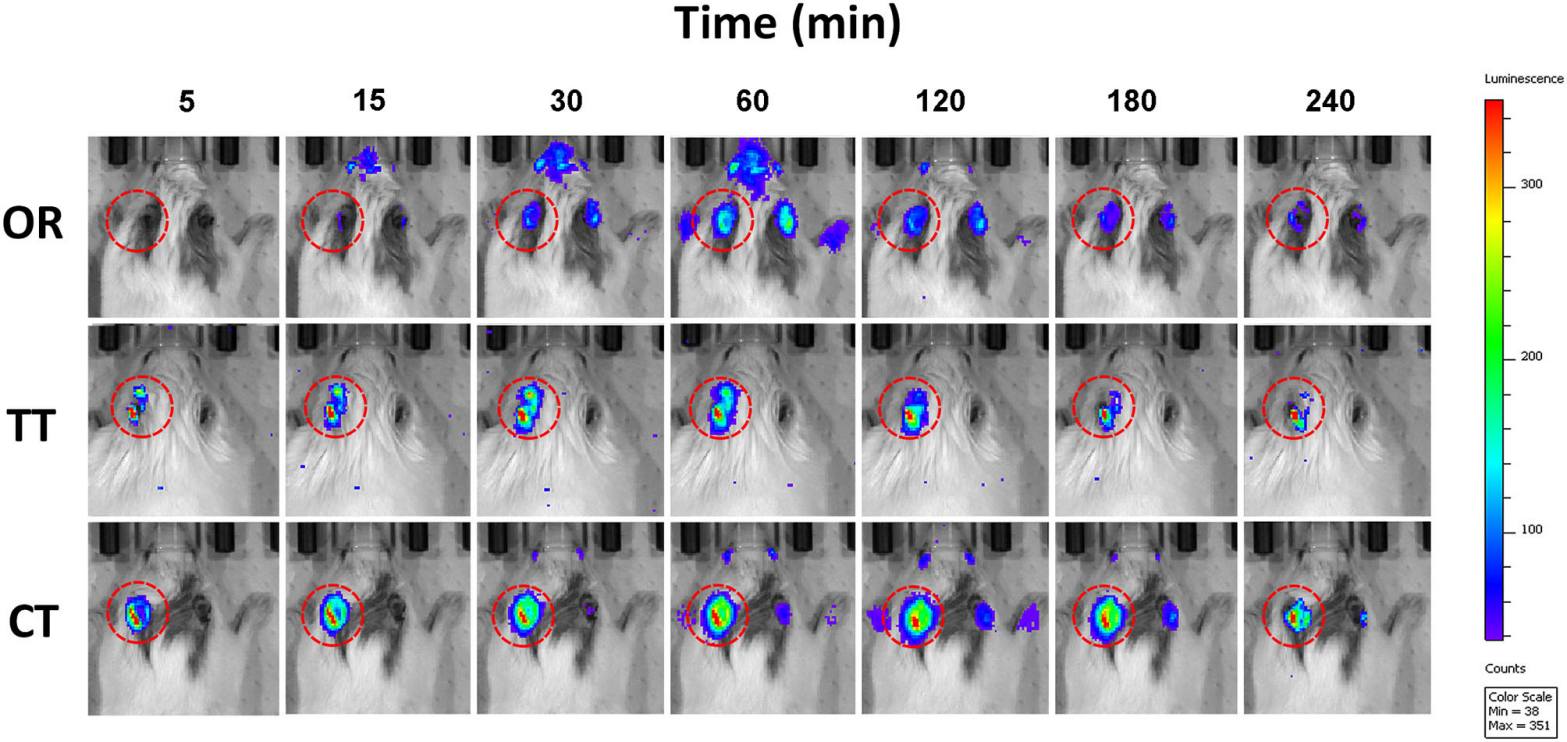
Photon bioluminescence over time course in *GFAP-Luc* mice given luciferin. Time course showing photon bioluminescence in the inner ears of mice in oral administration (OR), transtympanic administration (TT), and combined therapy (CT) groups.

Bioimaging revealed changes in the bioluminescence intensity over time (Figure 1). In the OR group, bioluminescence signals were observed in the inner ear 15 min after oral administration, reaching a peak in 60 min. Bioluminescence intensity gradually faded thereafter. In the TT and CT group, detectable bioluminescence appeared much earlier than that in the OR group. Very weak signals were detected in the right ears of OR group and CT group of mice.

**Figure 2:**
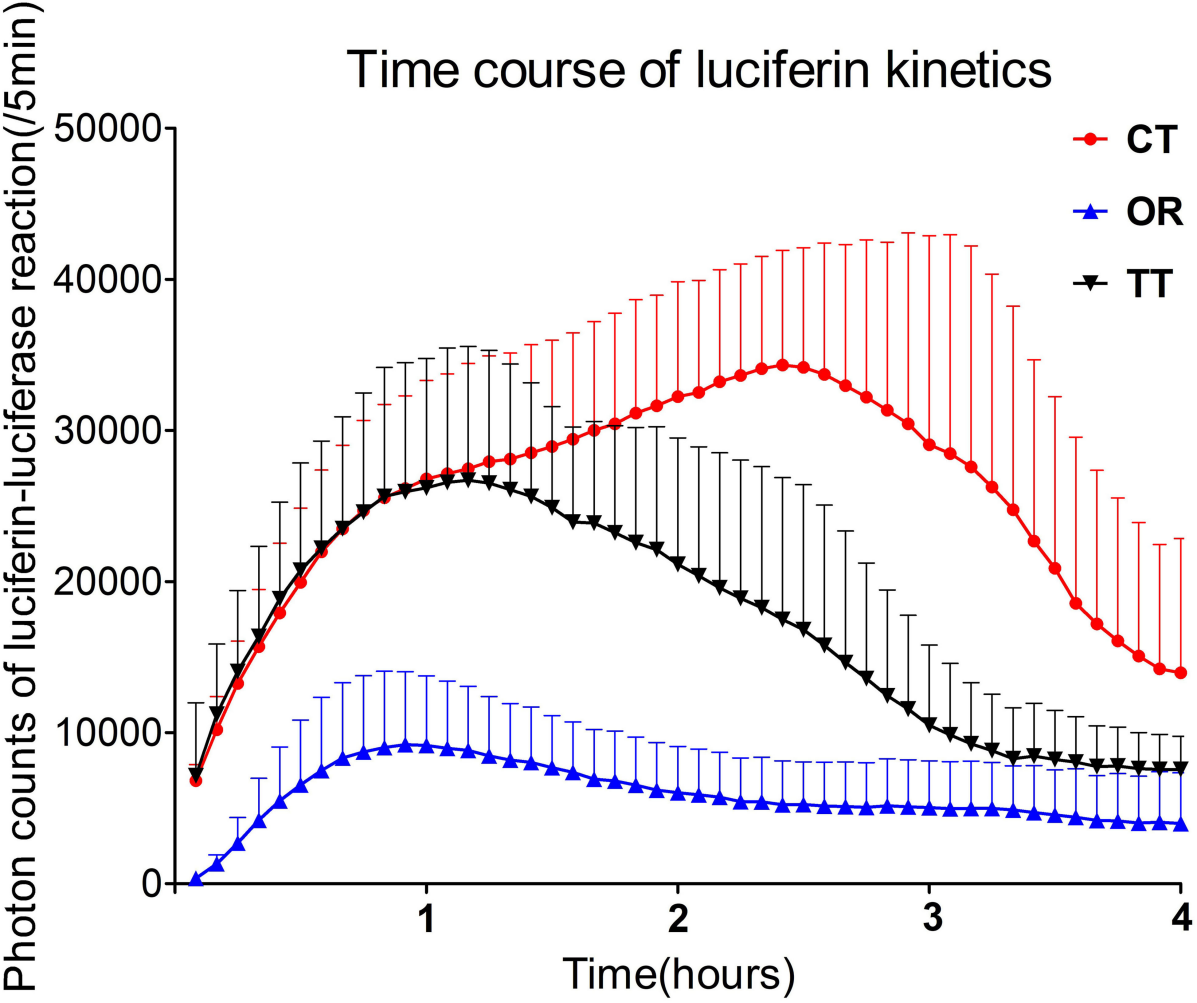
Time-dependent changes in photon counts of *GFAP-Luc* mice administered luciferin *via* different routes. Error bars indicate standard errors.

**Figure 3:**
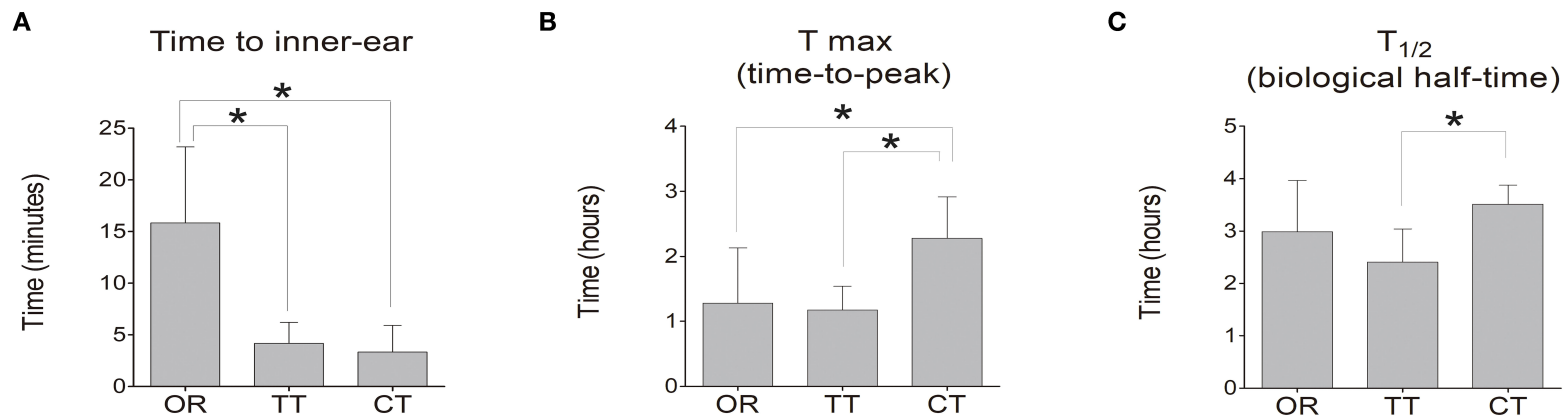
Time to inner-ear, time-to-peak of photon count (T_max_), and biological half-life (T_1/2_) of D-luciferin in the inner ear. **(A)** Time at which photon count appeared in the inner ear in each group; **(B)** T_max_ of D-luciferin was detected in each group. **(C)** T_1/2_. Data represent means ± SE. The bar graph shows the average value of the mice in each group, and the error bar indicates the standard deviation. *P < 0.05.

Luciferase kinetics were quantitatively evaluated for the time course in the inner ear (Figure 2) In the OR group, the time when bioluminescence signals could be observed in the inner ear was 15.8 ± 7.4 min, whereas this time was significantly reduced in the TT group (4.1 ± 2.0 min) and the CT group (3.3 ± 2.6 min) (*P* < 0.05 for each; *n* = 6) (Figure 3A). The maximum peak photon count was reached in 1.3 ± 0.9 h in the OR group and 1.2 ± 0.4 h in the TT group. In contrast, the peak time of the CT group was 2.3 ± 0.6 h, which was significantly longer than that of the other two groups (*P* < 0.05 for each; *n* = 6) (Figure 3B). The biological half- life was 3.0 ± 1.0 h in the OR group, 2.4 ± 0.6 h in the TT group and 3.5 ± 0.4 h in the CT group, and the half time of the CT group was longer than that of TT group (*P* < 0.05; *n* = 6) (Figure 3C).

**Figure 4:**
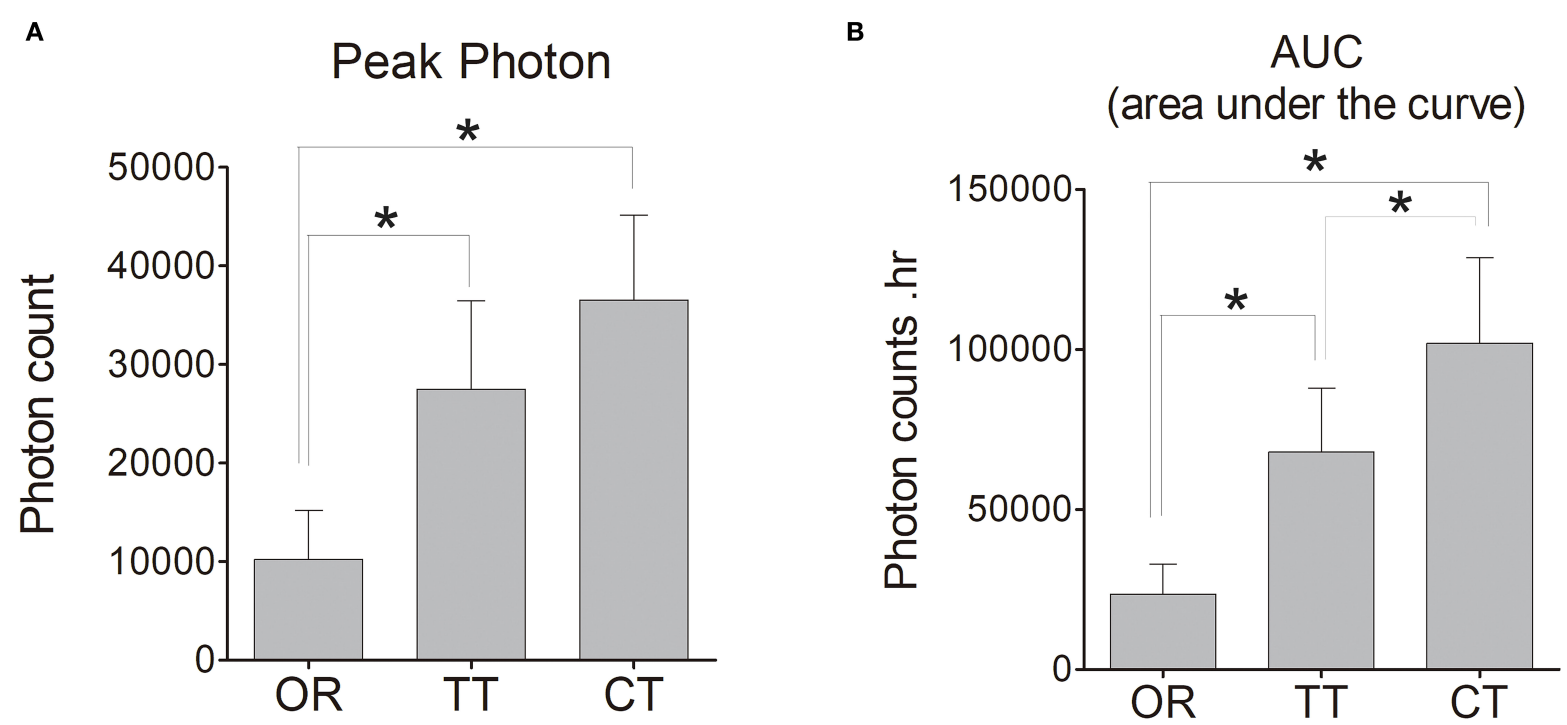
Peak photon count and the area under the curve (AUC) of D-luciferin in the left inner ear of each group of mice. **(A)** Peak photon; **(B)** AUC. The bar graph shows the average value of mice in each group, and the error bars indicate the standard deviation. *P < 0.05.

An analysis of pharmacokinetic values did reveal significant differences in the peak values between OR group and TT group and between OR group and CT group (*P* < 0.05 for each; *n* = 6). Compared to TT group, CT group did not displayed stronger peak value (Figure 4A). However, regarding AUC, CT group owned the largest area among these three groups, and significant differences existed between any 2 groups (*P* < 0.05 for each; *n* = 6) (Figure 4B).

## Discussion

Local drug delivery including intratympanic and intracochlear administrations has important advantages to treat inner ear disorders, offering precision of dose and avoidance of possible adverse effects associated with systemic administration (18). Intracochlear administration through an opening in the cochlear bony wall is more efficient than intratympanic administration but is rather invasive, and performed only during surgery in current clinical practice. For example, Prenzler NK used a cochlear catheter to inject the steroid into the cochlea and achieved the decrease of the electrical impedance during human cochlear implantation (19, 20). Although additional routes of delivery have been explored, including a comparison of round window injections vs. placement of drug-delivering materials on the surface of the round window membrane, intralabyrinthine injections to the semicircular canal or vestibule, and local activation following systemic delivery (21, 22), conventional oral route of administration, intratympanic injection of liquid solutions and combination of these two routes that we used in this study are still widely performed clinically and closely resemble the projected route of administration to humans.

The concentration and the volume of a drug should be considered when investigating pharmacokinetic effect of different delivery routes. The concentration of D-luciferin we used in this study is 15 mg/ml, the same as that of our previous studies (12–15), which suggest this concentration is safe and effective for systemic and local administration. Meanwhile, our previous study has demonstrated that it is the volume of D-luciferin delivered systemically, not its concentration that correlates with its inner ear pharmacokinetic (13). Therefore, we did not perform dose-response experiments to investigate the optimal concentration of D-luciferin in present study. Although the more volume of a drug applied systemically could increase the concentration of that drug in the inner ear (15), the recommended volume for the oral route of administration is 10 μl/g for mice because large dose volumes could overload the stomach capacity and reflux into the esophagus (23, 24). According to the body weights of mice from 26 to 34 g in this study, we used 280 μl volume to perform oral administration.

Our results showed that drugs need a longer time to enter into the inner ear by conventional OR route, compared with the TT or CT route. Although we used four times the volume of D-luciferin for OR administration, the peak photon and AUC value of the OR group were significantly lower than those in the other two groups. This result indicates that the OR route is largely ineffective because the blood-perilymph barrier reduces the exchange between the plasma and the inner-ear fluids. Very weak signals detected in the right ear after OR administration suggests its potential side effects. To achieve an effective therapeutic concentration in the inner ear, a larger volume or higher dose of the oral drug needs to be delivered, which may increase the systemic drug toxicity and the risk of related adverse effects.

TT is minimally invasive but relies on diffusion through the round window membrane for drug entry into the cochlea. Compared to oral administration, TT has the advantage of reducing systemic side effects during long-term application. The drug's residence time in contact with the round window should be sustained and controlled as closely as possible to increase the drug concentration in the inner ear and reduce drug level variability (18). In this study, the total study period reached 4 h, which ensured the drug-filled the middle ear cavity and made contact with the round window. We found that D-luciferin entered into the cochlea in about 5 min, reached a peak about 1 h after TT injection, and then gradually faded. This result is consistent with the findings of our previous studies (12, 15) and also implies that, in practice, patients need to maintain the position for 1 h after TT injection to achieve the highest drug concentration in the inner ear.

CT is regarded as a new therapeutic strategy for the treatment of ISSNHL (5). However, the efficacy of combination therapy for ISSNHL remains controversial (25, 26), and it has not yet been elucidated whether OR administration in combination with TT injection provides an additional advantage over OR or TT alone in patients with ISSNHL. In this study, the temporal-spatial distribution of a drug could be clearly monitored by the IVIS system. From the efficiency perspective, CT had a shorter onset time than OR, but longer reaching-peak time than TT, indicating luciferin could rapidly enter the inner ear, but reach peak value slowly after CT. From the efficacy perspective, CT had longer T1/2 time and higher AUC value than TT, implying more luciferin entering inner ear and longer residence time in CT group. Of note, there was no significant difference in peak value of D-luciferin in the inner ear between the TT and CT groups. This suggests that adding a quadruple volume of luciferin to TT by oral administration can deliver more drugs into the inner ear and confer a longer therapeutic window, but cannot increase drug intensity.

This study had certain limitations. Although luciferin is the only drug used for drug delivery detection in live animals, it differs from the steroids commonly used to clinically treat ISSNHL. Second, different doses and concentrations of drugs should be compared among the OR, TT, and CT routes. Concerning these points, further investigation and analysis is needed to address these limitations.

To conclude, the OR route is less effective than the TT or CT route, combination of OR and TT can deliver more drugs into the inner ear, and confer a longer therapeutic window, but cannot increase drug intensity.

## Data Availability Statement

The raw data supporting the conclusions of this article will be made available by the authors, without undue reservation.

## Ethics Statement

The animal study was reviewed and approved by Animal Care and Use Committee of Keio University (Permit Number: 08020). Written informed consent was obtained from the owners for the participation of their animals in this study.

## Author Contributions

YL and SK conceived the study, carried out the experiments, collected, and analyzed data. KO directed the project. SS and HO provided advice on the experiments. All authors participated in discussing the data.

## Conflict of Interest

The authors declare that the research was conducted in the absence of any commercial or financial relationships that could be construed as a potential conflict of interest.

## Publisher's Note

All claims expressed in this article are solely those of the authors and do not necessarily represent those of their affiliated organizations, or those of the publisher, the editors and the reviewers. Any product that may be evaluated in this article, or claim that may be made by its manufacturer, is not guaranteed or endorsed by the publisher.
